# Understanding undergraduate nursing students’ learning journeys with artificial intelligence: a journey mapping study

**DOI:** 10.1186/s12909-026-09271-y

**Published:** 2026-04-23

**Authors:** Luo Yang, Na Yue, Jianing Mao, Chen Huang, Dan Liu, Shanshan Chen, Yanyan Jiang, Yeqin Yang, Guijuan He

**Affiliations:** 1 School of Nursing, Zhejiang Chinese Medical University, 548 Binwen Road, Binjiang District, Hangzhou, Zhejiang 310053 China; 2 Institute of Higher Education, Zhejiang Chinese Medical University, 548 Binwen Road, Binjiang District, Hangzhou, Zhejiang 310053 China; 3 Department of Anesthesiology, Sixth Medical Center, PLA General Hospital, 6 Fucheng Road, Haidian District, Beijing, 100048 China; 4 Department of Nursing, Sir Run Run Shaw Hospital, Zhejiang University School of Medicine, 3 East Qingchun Road, Shangcheng District, Hangzhou, Zhejiang 310016 China; 5 Department of Adult Cardiac Surgery, Fuwai Hospital, Chinese Academy of Medical Sciences, 167 Beilishi Road, Xicheng District, Beijing, 100037 China; 6 Department of Nursing, Beijing Hospital, 1 Dahua Road, Dongcheng District, Beijing, 100730 China

**Keywords:** Artificial intelligence, Nursing education, Learning journey, Journey mapping, Undergraduate nursing students

## Abstract

**Background:**

As artificial intelligence (AI) becomes increasingly embedded in healthcare, nursing education faces growing expectations to prepare students with AI literacy. However, nursing students often encounter cognitive, emotional, and practical challenges when learning AI, and their learning experiences across the educational trajectory remain insufficiently understood. This study employed journey mapping to explore how undergraduate nursing students experience learning AI over time.

**Methods:**

A qualitative descriptive design was used. Seventeen undergraduate nursing students from four universities in different regions were purposively recruited between March and September 2025. Semi-structured interviews were conducted and analyzed using content analysis. A journey map was developed to integrate students’ learning tasks, emotional trajectories, barriers, and needs across different stages of AI learning.

**Results:**

The AI learning journey was characterized by four stages: Initial Contact and Curiosity, Learning and Confusion, Integration and Anxiety, and Internalization and Confidence. Across these stages, students experienced distinct and evolving challenges. Early learning was characterized by limited relevance and fragmented exposure to AI concepts. Subsequent stages involved cognitive overload, theory–practice gaps, ethical uncertainty, and career-related anxiety, while later stages highlighted the need for feedback, institutional support, and opportunities for innovation and professional identity consolidation.

**Conclusions:**

Journey mapping revealed the dynamic and stage-specific nature of nursing students’ AI learning experiences. These findings highlight the importance of stage-sensitive educational approaches that combine early cognitive guidance, scaffolded technical learning, ethical reflection, and innovation-oriented support to strengthen AI education in undergraduate nursing programs.

**Supplementary Information:**

The online version contains supplementary material available at 10.1186/s12909-026-09271-y.

## Introduction

With the rapid advancement of artificial intelligence (AI) and its increasing integration into the healthcare sector, smart healthcare has emerged as a new driving force for high-quality development in the health industry [1]. As an essential component of the healthcare system, nursing is undergoing profound transformations brought about by AI [2]. From intelligent health monitoring and clinical decision support to robot-assisted care, AI applications are reshaping both the practice scenarios and the underlying connotations of nursing [3]. This trend has placed new demands on future nursing professionals, particularly the ability to collaborate effectively with intelligent tools while understanding their underlying principles, strengths, and limitations, thereby cultivating essential AI literacy [4, 5]. Consequently, the systematic integration of AI education into undergraduate nursing programs has become increasingly important for preparing future nurses to engage with intelligent healthcare.

However, AI education in nursing remains at an exploratory and early stage compared with the pace of technological development [6]. Although many nursing schools in China have begun offering courses such as Intelligent Nursing and Nursing Informatics, numerous challenges persist in teaching practice. Existing studies have primarily focused on curriculum design, content development, or the effectiveness of specific teaching methods, while relatively few have explored AI learning from students’ perspectives across the learning process [7, 8]. This gap between teaching and learning perspectives may limit the alignment of educational reform with students’ actual learning needs. Therefore, there is an urgent need for a research tool capable of visually capturing students’ full learning experiences and identifying key challenges, thereby providing actionable insights for instructional improvement.

Journey mapping, a qualitative research method originating from service design, enables the visualization of users’ behaviors, touchpoints, thoughts, emotions, and pain points along a timeline, systematically uncovering needs and obstacles across the entire experience process [9]. Applying this method to educational contexts allows researchers to move beyond isolated courses or fragmented teaching episodes, examining students’ learning journeys from a dynamic, continuous, and holistic perspective.

Building upon this rationale, the present study employs the journey mapping approach to comprehensively explore the complete learning journey of undergraduate nursing students in the context of AI education. The study aims to identify stage-specific learning barriers, emotional fluctuations, and support needs across students’ AI learning journeys. The findings are expected to provide empirical insight for improving AI education in undergraduate nursing programs and advancing a more student-centered understanding of AI learning in nursing education.

## Methods

This qualitative descriptive study explored undergraduate nursing students’ learning experiences with AI and used journey mapping to visualize their evolving tasks, emotions, barriers, and needs throughout the learning process. The study follows the Consolidated Criteria for Reporting Qualitative Research (COREQ) 32-item checklist to ensure methodological rigor and reporting transparency [10].

### Design

A qualitative descriptive design was adopted to capture students’ authentic perceptions and learning experiences regarding AI education. Semi-structured individual interviews were used as the primary data collection method, while journey mapping served as an analytical and visualization framework for identifying key touchpoints and developmental patterns. The research team comprised nursing faculty and researchers trained in qualitative methods, with prior experience conducting semi-structured interviews and analyzing qualitative data. The interviewers (all female researchers) received additional training in reflexive interviewing, neutral probing, and bracketing strategies before data collection. They had no teaching or supervisory roles over the participants, ensuring no hierarchical relationships that might influence disclosure.

### Participants

Purposive sampling was used to recruit undergraduate nursing students from four universities in Hangzhou and Beijing, China, between March and September 2025. Participants from different institutions and academic stages were included to capture a broader range of AI learning experiences across the undergraduate trajectory. Inclusion criteria were: (1) full-time undergraduate nursing students; (2) having completed or currently enrolled in at least one course containing core AI-related content; (3) age ≥ 18 years; (4) able to communicate fluently and willing to articulate their experiences; and (5) voluntary participation with consent for audio-recorded interviews. Students who were on leave, unable to complete interviews, or who had insufficient course attendance to recall learning experiences clearly were excluded.

Recruitment occurred through class announcements and instructor referrals, but no pre-existing personal relationships existed between participants and interviewers. Interested students contacted the research team directly, and researchers provided detailed study information before obtaining written informed consent. Interviews were scheduled at participants’ convenience. Sample size was determined by data saturation, defined as the point at which no new themes or insights emerged from consecutive interviews [11]. Data saturation was assessed concurrently with data collection and analysis. Ethical approval for this study was obtained from the university’s ethics committee (Approval No. 20250523-2).

### Journey map construction

Journey mapping followed established procedures [12] and aimed to depict students’ evolving experiences along a temporal sequence. Journey mapping was not used merely as a visual presentation tool, but as an analytical framework that integrated temporal sequencing, emotional trajectories, learning tasks, and contextual barriers across the learning process. By mapping experiences along a chronological learning journey, this approach enabled the identification of stage-specific challenges and intervention points that may not be readily captured through traditional thematic analysis alone. In the context of health education research, this approach was considered particularly suitable because it enabled the analysis of how students’ learning experiences, needs, and emotional responses evolved over time within specific educational contexts.

Based on a literature review [13–15] and team discussions, an initial framework encompassing potential learning stages and experience dimensions was drafted. A descriptive qualitative approach [11] was then adopted to conduct semi-structured interviews with undergraduate nursing students, enabling the refinement and enrichment of the preliminary framework through authentic experiential narratives. During this process, the research team identified segments of interview data that reflected students’ experiences throughout the AI learning process and analyzed them for recurring patterns related to actions, emotional responses, challenges, support needs, and critical transitions. These coded segments were compared across participants and organized according to temporal sequence and experiential progression. Through iterative grouping and constant comparison, related codes were clustered into broader experiential patterns that represented meaningful shifts over time. Preliminary labels for these stage patterns and analytic dimensions were then developed inductively and refined through repeated team discussion, ongoing comparison with the original transcripts, and iterative review until consensus was reached on the overall journey structure.

As data collection and analysis progressed, the preliminary journey structure was iteratively refined through team discussions, peer debriefings, and comparison with emerging codes. To enhance the credibility and accuracy of the map, representative participants were invited to review the draft journey map and provide feedback on its completeness and resonance with their lived experiences. Their feedback was used to examine whether the phase structure, key elements, and overall interpretation adequately reflected students’ actual learning experiences, and this process informed the final refinement of the journey map.

### Development of the interview guide

The semi-structured interview guide was developed based on the study aims, a review of relevant literature, and preliminary observations of students’ learning contexts. Two experts in nursing education reviewed the initial guide to assess content validity and ensure that the questions aligned with the conceptual focus of the study. A pilot test with two non-participant nursing students was then conducted to evaluate clarity, relevance, and comprehensibility. Minor modifications were made to improve wording, logical flow, and ease of understanding.

The final interview guide consisted of open-ended questions addressing students’ initial perceptions of AI, learning motivations, cognitive and emotional challenges, connections between theoretical knowledge and clinical practice, and perceived barriers or facilitators encountered throughout their AI learning journey. During data collection, the guide was used flexibly; probing and follow-up questions were introduced when needed to explore emerging issues in greater depth and to ensure richness and completeness of participants’ narratives. The full interview guide is provided in Supplementary File 1.

### Data collection

All interviews were conducted face-to-face by trained interviewers in settings chosen to maximize comfort, privacy, and feasibility, including quiet rooms on campus, meeting rooms in affiliated hospitals, or secluded areas of local cafés. Regardless of location, efforts were made to ensure confidentiality and minimize interruptions. At the beginning of each interview, the interviewer engaged in rapport-building, explained the study purpose and confidentiality procedures, and emphasized voluntary participation to foster a trusting environment. Only the interviewer and participant were present during each session. The interviews were conducted by three female researchers who had received structured training in qualitative methods.

Participants were encouraged to speak freely, with the interviewer maintaining a neutral stance and avoiding suggestive or leading questions. Field notes were taken during and immediately after each interview to capture contextual details, nonverbal expressions, and researcher reflections.

Interviews generally lasted between 25 and 45 min, with an average duration of approximately 27 min, and were audio-recorded with participants’ consent. Four participants were invited for follow-up interviews to clarify or expand on emerging themes. Data collection continued until ongoing analysis indicated that data saturation had been achieved. No financial incentives were provided.

### Data organization and analysis

Audio recordings were transcribed verbatim within 24 h. NVivo 14.0 software was used to manage and organize the data. Analysis followed the steps of traditional content analysis [16]: (1) repeated reading of transcripts; (2) extraction of meaningful units; (3) open coding; (4) grouping codes into categories; and (5) abstraction of themes aligned with the journey map structure. Three researchers independently coded the initial transcripts, compared coding outcomes, and resolved discrepancies through discussion to enhance analytic consistency. The preliminary coding framework was then iteratively applied, reviewed, and refined as analysis progressed across the full dataset.

An audit trail was maintained documenting coding decisions, analytic memos, meeting notes, and iterative versions of the journey map. Triangulation was achieved through multiple coders, team debriefings, and participant validation. Specifically, multiple researchers were involved in coding and analysis, and iterative team debriefings were conducted to compare interpretations and refine emerging categories. Participant validation was carried out by inviting representative participants to review the draft journey map and preliminary findings and to comment on whether these interpretations were consistent with their learning experiences. These procedures enhanced the credibility of the findings and reduced the risk of relying on a single researcher’s interpretation. The integration of emotional trajectories and learning needs strengthened the interpretive depth of the journey map. Representative quotes from participants are presented in the results to support thematic interpretations, with anonymized identifiers (N1–N17).

### Researcher reflexivity and methodological rigour

In line with COREQ principles, methodological rigor was ensured through reflexivity, credibility checks, and transparent reporting. The research team continuously reflected on their assumptions and potential biases through memo writing and discussion. Because the researchers had backgrounds in nursing education and qualitative research, and were familiar with the topic of artificial intelligence in nursing education, they recognized that their prior knowledge and positive interest in the field might shape the interpretation of participants’ accounts. To minimize this potential influence, the team maintained reflexive memos throughout the analytic process, critically discussed preliminary interpretations, and repeatedly returned to the original transcripts to ensure that the findings remained grounded in participants’ narratives rather than researchers’ expectations. Credibility was strengthened through participant validation, independent coding, and iterative peer debriefings. Dependability was enhanced by maintaining an audit trail detailing analytic procedures and decision-making steps. Transferability was addressed by providing thick descriptions of participants, context, and learning environments. Confirmability was supported through reflexive documentation, team discussion, and repeated checking of interpretations against the original data. Confidentiality and anonymity were rigorously protected throughout transcription, storage, analysis, and reporting.

## Results

A total of 17 nursing students were finally included in the study. The general demographic information of the participants is presented in Table 1.

**Table 1:** General Information of Participants (*n* = 17)

ID	Gender	Age (years)	Grade	Place of Origin	Prior AI Knowledge	AI-related Courses Completed (*n*)
N1	Female	20	Sophomore (Year 2)	Urban	Yes	1
N2	Female	21	Sophomore (Year 2)	Urban	No	2
N3	Female	20	Sophomore (Year 2)	Urban	No	1
N4	Male	20	Sophomore (Year 2)	Rural	No	1
N5	Female	20	Junior (Year 3)	Urban	No	1
N6	Male	21	Junior (Year 3)	Urban	Yes	2
N7	Male	22	Junior (Year 3)	Urban	No	1
N8	Female	21	Junior (Year 3)	Rural	No	1
N9	Female	20	Junior (Year 3)	Urban	No	2
N10	Male	21	Junior (Year 3)	Urban	No	1
N11	Male	21	Senior (Year 4)	Rural	No	1
N12	Female	22	Senior (Year 4)	Urban	Yes	1
N13	Female	23	Senior (Year 4)	Rural	No	1
N14	Female	22	Senior (Year 4)	Urban	No	2
N15	Male	22	Senior (Year 4)	Urban	No	3
N16	Female	22	Senior (Year 4)	Urban	Yes	1
N17	Female	21	Senior (Year 4)	Rural	No	1

**Fig. 1:**
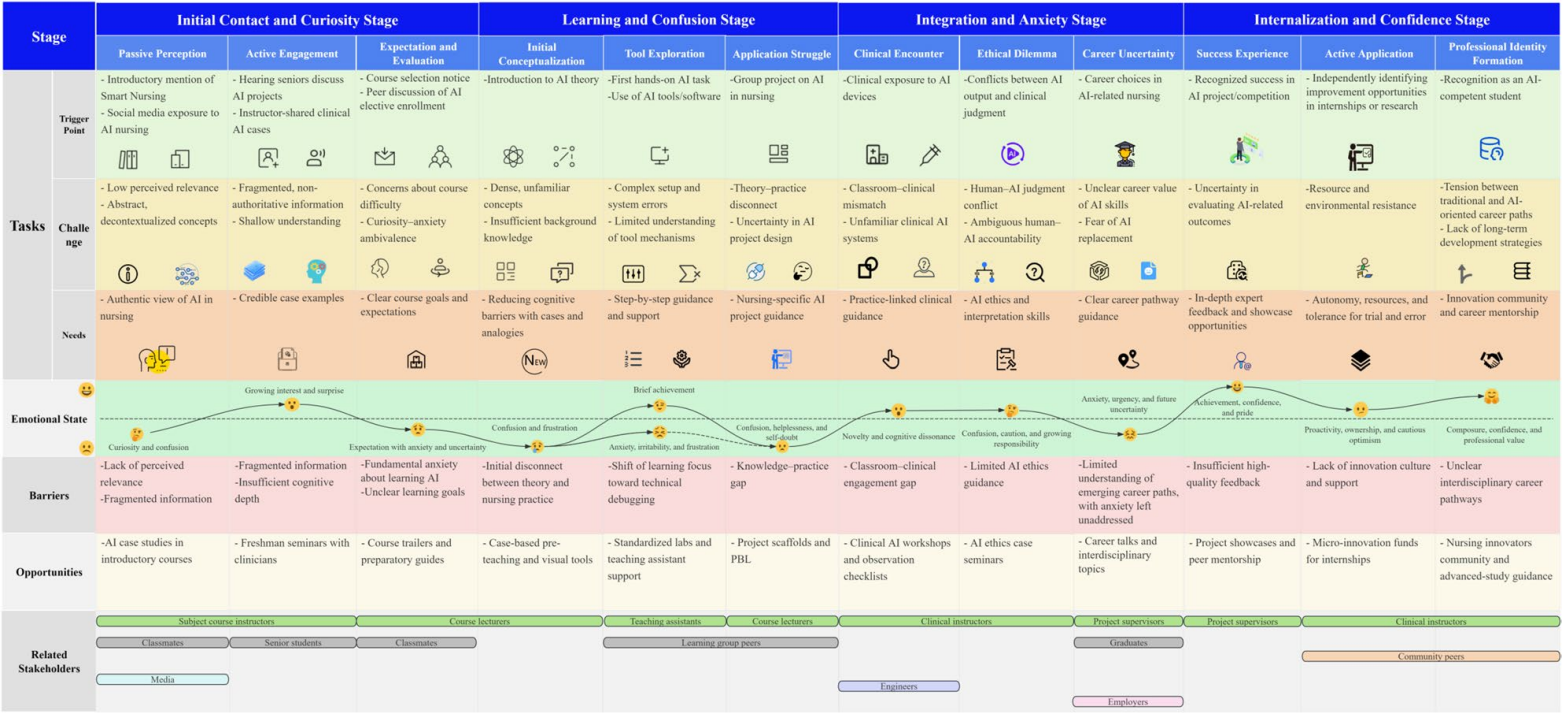
Journey map of undergraduate nursing students’ AI learning experiences

A total of 21 interviews were conducted in this study. Thirteen participants were interviewed once, while participants N2, N5, N7, and N13 participated in a second interview to ensure the completeness of data collection. The cumulative interview duration was 576 min, yielding 42,986 words of transcribed text. Based on the preliminary journey framework, the interview data were further analyzed in depth. Across different stages of the AI learning journey of undergraduate nursing students, 147 initial codes were generated and categorized into 24 themes (six themes for each stage) from three analytical dimensions: tasks, emotions, and pain points. These themes captured the nursing students’ experiences at each stage, including their touchpoints, needs, emotional fluctuations, challenges, and opportunity points. The journey map was developed to visualize the multi-dimensional storyline across all stages of the learning journey (see Fig. 1).

A journey map typically consists of a horizontal axis (timeline) and a vertical axis (task dimension) [12]. In this study, the horizontal axis of the nursing students’ AI learning journey was divided into four stages: (1) Initial Contact and Curiosity Phase, (2) Learning and Confusion Phase, (3) Integration and Anxiety Phase, and (4) Internalization and Confidence Phase. The horizontal axis was constructed based on the nursing students’ learning experiences and key activities across different stages. The vertical axis was developed according to the research objectives and included major elements in the students’ journey, such as triggers, challenges, needs, emotions, pain points, opportunities, and related stakeholders.

### Initial contact and curiosity stage

In this stage, students were first systematically exposed to the concept of AI. However, due to the lack of intuitive understanding within nursing-specific contexts, they found it difficult to establish a meaningful connection between AI and their future professional roles [17]. In addition, fragmented information sources further limited their understanding, resulting in superficial cognitive awareness [18]. Overall, students demonstrated mixed emotions of curiosity and confusion during this initial phase.

#### Passive perception

At the beginning of their undergraduate studies, nursing students passively received information about AI through introductory professional courses and media reports. However, most perceived AI as belonging to the computer science domain and considered it irrelevant to nursing practice. As one participant noted:

*“At that time*,* I thought AI was like robots or other high-tech stuff. It seemed related to computer majors*,* not something nurses would actually use.”* (N3)

Another student shared a similar impression:

*“Our teacher briefly mentioned smart nursing in class*,* but it felt distant from us. What came to mind were still injections and medications.”* (N7)

Moreover, the information they encountered was often abstract and fragmented, preventing the formation of accurate understanding.

*“The news always talks about how powerful AI is*,* but I have no idea what it really is or how it works.”* (N5)

*“It’s just some vague concepts to me. I’ve heard of them but didn’t form any deep impression.”* (N12)

#### Active engagement

When exposed to concrete examples of AI applications in nursing through peer sharing or course case studies, students began to develop interest and actively seek further information.

*“When I heard a senior student talking about an AI project predicting fall risks*,* I suddenly realized that this could actually be used in nursing—it became interesting to me.”* (N11)

*“The teacher showed a video of AI-assisted diagnosis in class*,* and it looked amazing. I even searched for more information afterward.”* (N8)

At this stage, students’ understanding of AI started to form initial connections with nursing practice [19].

*“I learned that AI can help with decision support and health monitoring. It made me think nurses could work more efficiently and accurately in the future.”* (N14)

*“I began paying attention to news about AI and would discuss it with classmates when I saw something interesting.”* (N2)

#### Expectations and trade-offs

Before officially enrolling in AI-related courses, students weighed the course difficulty, content, and their own academic foundations, experiencing emotions oscillating between anticipation and anxiety [20].

*“I hesitated before choosing Intelligent Nursing. I felt it’s a trend worth learning*,* but I was afraid my weak math and poor computer skills would make it hard to keep up.”* (N9)

*“I was curious about what exactly we would learn. I hoped it wouldn’t be all theory—maybe we could actually do some small projects ourselves.”* (N1)

While students acknowledged the value of such courses, they expressed a strong desire for clear learning pathways and guidance to reduce uncertainty and anxiety.

*“I know this course is important*,* but I wish the teacher could explain clearly in the first class what we’ll learn*,* how we’ll learn it*,* and what it’s useful for.”* (N6)

*“If we were told in advance what basic knowledge we need*,* we could review on our own and wouldn’t feel so nervous.”* (N10)

### Learning and confusion stage

In this stage, students began to systematically learn the theories and technologies of AI. However, due to the abstract nature of the course content, the complexity of technical operations, and the difficulty in linking AI with nursing scenarios, they generally experienced significant cognitive load and practical challenges. The predominant emotions during this phase were confusion, frustration, and uncertainty [21].

#### Initial exposure to concepts

At the beginning of the course, students were introduced to abstract concepts and mathematical principles such as machine learning and deep learning. Due to their limited prior knowledge, most students found it difficult to comprehend the material and experienced cognitive overload.

*“The teacher covered so many models and formulas in one class—it felt like listening to a foreign language. I couldn’t keep up at all and felt really frustrated.”* (N5)

*“All those algorithm names sound similar and confusing. I just couldn’t remember or tell them apart.”* (N8)

The disconnect between theoretical knowledge and nursing practice began to emerge at this stage, which further weakened students’ learning motivation.

*“I kept wondering what all these formulas and models could possibly do in a hospital setting. They seemed far removed from real nursing work.”* (N12)

*“It all felt so abstract. I couldn’t see the real meaning of learning this—just something to get the credits.”* (N1)

#### Tool exploration

During the practical sessions, students were required to operate specific AI tools or programming languages, yet technical barriers emerged as the primary challenge. A substantial amount of learning time was consumed by software installation, environment configuration, and troubleshooting errors.

*“It took me several days just to install the software and various libraries. Errors kept popping up*,* and I almost broke down before even getting to the AI part.”* (N4)

*“I followed every step in the lab manual*,* but the result just wouldn’t show up. I couldn’t find out why*,* and it made me really anxious.”* (N11)

The “black-box” nature of tool operations made it difficult for students to understand the underlying principles, thereby weakening the learning effectiveness.

*“It felt like just clicking buttons according to the steps. I could get the results*,* but had no idea why or what happened in between.”* (N7)

*“It’s like memorizing procedures. Once the dataset or problem changes*,* I can’t do it anymore—I haven’t really learned it.”* (N15)

#### Struggling to make connections

When the course required students to complete a project integrating AI technologies with nursing scenarios, they encountered their greatest challenge—applying theoretical knowledge and technical skills to solve real nursing problems, revealing a pronounced “knowledge–practice gap.”

*“The teacher asked us to choose a nursing problem and use AI methods to solve it. Our group had many ideas*,* but we got stuck on how to ‘translate’ a nursing problem into data and models that AI could process. We had no clue.”* (N2)

*“I know logistic regression can be used for classification*,* but how do I apply it to predict pressure ulcer risk? What kind of data do I need? How do I select the features? I was totally confused.”* (N9)

At this stage, feelings of frustration and self-doubt were particularly strong.

*“It felt like all the previous lessons were useless—the theory and practice didn’t connect. When it came time to actually use it*,* I realized I couldn’t do anything. It was really discouraging.”* (N6)

*“The ideas were good*,* but implementing them was so difficult that I started doubting whether I was suited for this at all.”* (N13)

### Integration and anxiety stage

At this stage, students entered clinical observation or internship placements, where they encountered and applied AI technologies in real healthcare settings for the first time. The idealized classroom understanding of AI often clashed with the complexities of clinical reality, triggering students’ deep reflections on technological limitations, ethical responsibilities, and their own professional futures, accompanied by a sense of anxiety and uncertainty [22].

#### Clinical validation

In the clinical setting, students witnessed the real-world application of AI tools and found that their actual roles and modes of operation often differed from classroom expectations. Their understanding gradually shifted from idealized to realistic perceptions.

*“When I saw the intelligent alert system in the ward*,* I realized that nurses mainly follow preset procedures*,* and AI decisions are only for reference. The final judgment still depends on the nurse’s experience—it wasn’t as ‘disruptive’ as what we learned in class.”* (N7)

*“The systems used in hospitals were much older and simpler than those introduced in class. It felt like theory and reality were somewhat disconnected.”* (N3)

At this stage, students also experienced a sense of unfamiliarity and distance when operating clinical systems due to their lack of hands-on experience.

*“The instructor handled the system so skillfully*,* but when it was our turn*,* we hesitated to try. I was afraid of pressing the wrong button and couldn’t really explain how it worked.”* (N9)

#### Reflective critique

When students observed or personally experienced discrepancies between AI-generated judgments and actual clinical situations or individual patient differences, they began to engage in critical reflections on the reliability and ethics of technology.

*“Once*,* the system issued a drug allergy alert*,* but the record was incorrect. That’s when I realized we shouldn’t blindly trust machines—nurses’ verification is still crucial.”* (N16)

*“The AI judged that the patient’s risk level was low based on the indicators*,* but the patient looked really unwell. I was confused—should I trust the data or my own clinical observation?”* (N5)

As a result, students developed deep confusion about the boundaries of responsibility in AI-assisted practice.

*“If we follow the AI’s suggestion and something goes wrong*,* who’s responsible—the nurse or the developer? This question was never discussed in class.”* (N14)

#### Future projection

As they approached graduation and employment, students felt the impact of AI technologies on the nursing profession, leading to uncertainty and anxiety about their future roles within the evolving healthcare ecosystem.

*“At the job fair*,* many hospitals mentioned smart hospital initiatives. I felt it was an opportunity*,* but also worried that my traditional nursing skills might lose value.”* (N2)

*“I’ve read articles saying AI might replace some nursing tasks*,* and that made me really anxious. I don’t know which skills I should focus on now to avoid being left behind.”* (N8)

Students expressed a strong desire for clear career guidance to help them cope with the uncertainty about the future.

*“I really want to know if there are specialized roles like ‘nursing informatics specialists’ and what new skills are required—right now I feel completely lost.”* (N6)

*“I hope the school can offer career planning guidance*,* showing us how to turn our AI knowledge into real professional competitiveness*,* instead of just talking about trends in general terms.”* (N12)

### Internalization and confidence stage

At this stage, students began to integrate AI knowledge and skills with their professional nursing identity. A few students who achieved positive outcomes through key projects or practical experiences demonstrated a shift from passive learning to active exploration and innovation. They gradually developed an emerging sense of professional identity as a new generation of nursing practitioners with AI literacy, accompanied by feelings of confidence, composure, and self-value.

#### Successful experiences

When students successfully completed a challenging AI-related project or validated the effectiveness of their ideas in practice, they developed a strong sense of self-efficacy, marking a key turning point in building confidence.

*“Our group’s graduation project used a machine learning model to predict fall risks among elderly inpatients. When the model showed high accuracy on the test set*,* the sense of accomplishment was unprecedented—it felt like we had truly achieved something.”* (N12)

*“During my internship*,* I suggested to the head nurse a small program to simplify health record data entry. After it was adopted*,* the efficiency really improved. At that moment*,* I realized what I had learned was genuinely useful.”* (N13)

At this stage, students expressed a desire for greater professional recognition of their achievements.

*“I hope that after the project ends*,* experts can give us more in-depth feedback—like how we could optimize it further*,* or whether it’s good enough to publish a paper. I want to know where I actually stand.”* (N10)

#### Active application

After accumulating successful experiences, students were no longer satisfied with merely completing assigned tasks. Instead, they began to proactively identify areas for improvement within clinical or learning environments and apply AI-oriented thinking to propose solutions.

*“During my pediatrics internship*,* I noticed that pain assessment mainly relied on subjective judgment. I wondered if facial expression analysis using image recognition could help*,* so I reviewed the literature and discussed a preliminary idea with my supervisor.”* (N17)

*“Now*,* when I see repetitive documentation or processes*,* I can’t help but think*,* ‘Could technology optimize this part?’ I start to actively consider the possibilities.”* (N5)

At this stage, students demonstrated a strong sense of ownership and initiative, though they also faced practical constraints in implementation.

*“I have good ideas*,* but it’s hard to move forward without data and institutional support.”* (N14)

#### Identity formation

Successful practical experiences and positive feedback encouraged students to internalize the identity of being “nurses who understand AI” as part of their self-concept. They were able to view technology more rationally and began to form clear and positive plans for their future professional development.

*“Now I feel that AI is a powerful tool*,* but the core still lies in nurses’ clinical thinking and humanistic care. My strength is that I understand both clinical work and technology—I can serve as a bridge between the two.”* (N15)

*“During job interviews*,* I highlighted my AI project experience*,* and the interviewers were very interested. I feel that ‘nursing + AI’ is my core competence and future direction.”* (N9)

Students also began to seek a sense of community belonging and to envision long-term professional growth.

*“I hope more classmates and teachers can join discussions and form a network. I’m even considering pursuing a master’s degree in nursing informatics to go deeper in this field.”* (N7)

## Discussion

### Cognitive bias and lack of instructional guidance as primary barriers to establishing students’ initial motivation for AI learning

During the “Initial Contact and Curiosity” stage, undergraduate nursing students often reported passive exposure to AI and a sense of confusion. Early encounters were typically triggered by brief mentions in introductory courses or fragmented representations in social and mass media. However, such exposure was frequently broad, decontextualized, and weakly connected to nursing practice [19, 23]. As a result, students struggled to situate AI knowledge within a professional nursing context [24], and some developed cognitive biases—viewing AI as “profound but impractical” or “irrelevant to nursing” [25, 26]. These perceptions were reinforced by the lack of systematic, guided instructional design in current curricula, which limited students’ opportunities to form an intuitive and practice-oriented understanding of “AI + Nursing” [20, 24]. Over time, the combined effects of low perceived relevance and fragmented information contributed to emotional detachment rather than sustained curiosity [27], making it more challenging for students to sustain long-term learning motivation.

The journey map suggests that proactive, early-stage interventions may help reshape students’ initial cognitive frameworks. Prior studies indicate that early instructional support can strengthen nursing students’ beliefs and self-efficacy regarding AI learning [28, 29]. One practical approach is to embed AI-related teaching within early professional induction courses (e.g., Introduction to Nursing) by integrating authentic nursing-relevant cases—such as risk assessment, diagnostic assistance, and rehabilitation guidance—to replace abstract descriptions and increase perceived relevance [30, 31]. In addition, freshman seminars featuring clinical experts and senior students may enhance students’ realistic understanding of AI applications and stimulate intrinsic motivation [21, 32]. Providing a structured course preparation guide before formal AI modules—clarifying learning objectives and prerequisite knowledge—may further support psychological readiness, set realistic expectations, and reduce anxiety, thereby improving engagement [33]. Collectively, such early-stage guidance may foster a more positive learning mindset and strengthen students’ resilience across subsequent stages of AI learning [34].

Beyond the early-stage barriers described above, teacher-related factors appeared to shape students’ learning experiences across multiple stages of the journey. Participants repeatedly linked their initial expectations, ongoing confidence, and willingness to persist with the clarity of instruction, the design of learning activities, and the emotional climate fostered in class. Insufficient early guidance, limited scaffolding for technical tasks, and constrained feedback opportunities were described as amplifying uncertainty and frustration. These findings align with international evidence that educators’ digital literacy, pedagogical strategies, and attitudes toward AI influence students’ motivation and perceived ability to develop AI literacy [35, 36]. Strengthening faculty development in AI literacy, curriculum design, and student-centered scaffolding may therefore be important for sustaining high-quality AI education in nursing.

### Practical barriers and the theory–practice gap as core drivers of learning frustration

The “Learning and Confusion” stage represented the period of greatest cognitive demand and emotional strain in students’ AI learning journey. Students described difficulty engaging with abstract concepts such as algorithms and data modelling, which often exceeded their prior knowledge base and contributed to cognitive overload [37]. In parallel, limited timely support for tool use or programming tasks created operational barriers, undermining self-efficacy and contributing to frustration and anxiety [38]. When the curriculum progressed to project-based tasks, the theory–practice gap became particularly salient: although students could understand AI principles conceptually, many struggled to translate these principles into nursing-relevant applications [39]. This disconnect contributed to cycles of uncertainty and self-doubt [40]. Overall, the core challenge at this stage appeared to be insufficient integration across conceptual learning, technical practice, and contextual application, resulting in a fragmented learning experience [41].

Students’ learning experiences with artificial intelligence may differ according to the type of tool they engage with. Generative AI tools, which provide rapid feedback, content generation, and interactive support, may stimulate curiosity, novelty, and short-term confidence, but may also raise concerns about accuracy, overdependence, and appropriate use [42, 43]. By contrast, more analytical or task-oriented AI tools often require stronger abilities in information interpretation, judgment, and contextual application, thereby creating different forms of cognitive challenge and emotional strain. This distinction helps explain why students’ emotional “peaks and valleys” were not entirely uniform across the learning journey [42]. From a theoretical perspective, the learning resistance identified in this study should not be interpreted simply as reluctance to adopt new technology. Rather, it may reflect a transitional challenge in the development of digital literacy, especially when students encounter unfamiliar tools, limited instructional support, or uncertainty about how AI relates to nursing practice [44]. In this sense, learning resistance may indicate difficulties in understanding, evaluating, and appropriately applying digital tools within a professional context, rather than mere unwillingness to engage [43, 44].

Addressing these challenges may benefit from a more coherent instructional pathway and supportive learning infrastructure. Instructional design could prioritize reducing cognitive load, strengthening contextual relevance, and enhancing self-efficacy [45]. For example, a “case-first, concept-later” approach—introducing nursing-relevant AI applications before explaining underlying principles—may help connect abstract concepts to practice. At the learning-environment level, accessible technical support (e.g., teaching assistants, peer collaboration, and timely feedback) may reduce operational barriers and support persistence [46]. Project-based learning may also provide a structured route for students to connect technical skills with nursing problems, thereby supporting transfer from conceptual understanding to contextual application [47]. Together, these strategies may offer integrated support across cognition, skills, and emotional regulation, facilitating sustained engagement with AI learning.

### Clinical integration challenges and future anxiety highlight the need for ethical education and career guidance

The findings of this study show that, during the “Integration and Anxiety” stage, nursing students’ learning challenges extended beyond classroom-based knowledge and skill acquisition to encompass more complex dimensions of clinical practice, ethical reflection, and professional identity formation. Upon entering clinical settings, students encountered for the first time the “non-idealized” reality of AI in nursing practice—where AI functioned primarily as an assistive tool rather than a decision-maker. This contrasted with classroom narratives that may sometimes frame AI as highly autonomous or transformative, contributing to cognitive dissonance. Students were required not only to understand the practical functions and limitations of AI within a safe and controlled environment, but also to learn how to balance technological reliance with humanistic judgment in the face of clinical complexity [48, 49]. When discrepancies arose between AI-generated recommendations and nursing experience, ethical ambiguity and blurred boundaries of responsibility became evident, leading many students to question whether technology should—or could—replace human judgment. This tension between ethical uncertainty and professional accountability shifted students’ learning focus from how to use AI toward how to coexist with AI appropriately. Meanwhile, career-related anxiety began to surface. As AI technologies rapidly permeate healthcare, students expressed uncertainty regarding their future roles, the value of traditional skills, and viable career trajectories, deepening concerns about occupational replacement and restricted professional growth [50]. The interplay of technological anxiety, ethical conflict, and career ambiguity reveals a systemic gap within current AI nursing education—specifically in areas of clinical adaptation, ethical guidance, and career development. As Johnson and Galatzan [51] emphasized, nursing education in the AI era must move beyond technical training, offering cognitive, emotional, and ethical support to help students construct a dynamic and resilient professional identity.

These findings also resonate with broader international discussions on technological competence and digital capability in nursing education [31]. Recent scholarship has increasingly emphasized that preparing future nurses for AI-enabled healthcare requires more than basic technical exposure; it also involves the development of digital understanding, critical judgment, ethical awareness, and the ability to apply technological tools appropriately in clinical contexts [31, 48]. From this perspective, the uncertainty described by students in the present study reflects not only difficulties in using AI-related tools, but also the broader developmental demands of becoming technologically capable nursing professionals [31]. In addition, the educational implications may differ according to the type of AI tool involved. Generative AI tools may primarily raise concerns related to accuracy, dependence, and ethical boundaries, whereas more analytical or task-oriented AI applications may place greater demands on interpretation, decision-making, and contextual integration [52]. Differentiating these tool types may therefore help explain the varied forms of challenge experienced by students and support more targeted approaches to AI learning in nursing education.

An important interpretive consideration is that students’ reports of encountering “AI technologies” in clinical placements often referred to broader digital or information systems rather than AI-driven tools. This conflation highlights a knowledge boundary in students’ understanding of digital health technologies and may partly explain the mismatch between classroom expectations and clinical observations. Explicitly differentiating AI from general information systems within curricula may therefore help students develop more accurate and realistic expectations regarding AI-enabled nursing practice.

To address these challenges, a multi-component educational approach that integrates ethical literacy, clinical exposure, and career guidance may be beneficial. First, learning opportunities could extend beyond classrooms through authentic or simulated clinical experiences that allow students to explore the capabilities, limitations, and safety considerations of AI-enabled systems [53]. Second, ethics content can be integrated using role-play, case-based debates, and reflective discussions to strengthen ethical reasoning and human–AI collaboration in complex decision contexts [54]. In parallel, structured career guidance—such as exposure to interdisciplinary pathways in nursing informatics and discussions with clinical leaders—may help students reframe anxiety as direction and motivation for continued learning [55].

Through such multi-dimensional interventions integrated across the entire educational continuum, educators can effectively support students in maintaining ethical awareness and professional confidence when facing the realities of AI in healthcare—facilitating their transformation from learners to intelligent nursing practitioners.

### Successful internalization and identity formation depend on supportive innovation environments and structured advancement pathways

The findings of this study indicate that nursing undergraduates who successfully reached the “Internalization and Confidence” stage often experienced a deep transformation from technical mastery to professional identity formation during their AI learning process. Their learning triggers typically stemmed from milestone experiences of success, such as completing a high-quality AI innovation project or effectively validating algorithmic solutions in clinical practice. These positive experiences significantly strengthened students’ self-efficacy and sense of accomplishment, encouraging them to view AI as an integral component of professional growth and nursing innovation [56]. However, as learning progressed, students encountered new advanced-level challenges, consistent with previous research [57]. Specifically, their innovative outcomes lacked systematic feedback and showcase platforms; creative ideas rarely received adequate resource support or faculty mentorship; and at the career development stage, students faced a shortage of professional communities and clear advancement pathways. These gaps may leave high-performing students without sustained feedback, mentorship, or community support, potentially weakening their longer-term motivation and identity consolidation [58]. Moreover, in the absence of long-term incentive mechanisms and sustained peer or community support, students’ innovative momentum and identity stability tend to fluctuate [59]. Thus, the core challenge of this stage lies in the absence of a sustainable support structure for cultivating innovative nursing talents in AI. Without such systems, successful experiences fail to accumulate, innovative outcomes remain underutilized, and career growth lacks depth and continuity, ultimately hindering the transition from “learning innovation” to “leading innovation.”

Strengthening institutional supports may help consolidate students’ internalization and emerging professional identity related to artificial intelligence in nursing practice. Regular opportunities to showcase student work (e.g., innovation forums or exhibitions) may enhance recognition and provide role models for earlier-stage learners. Small-scale seed funding, access to mentorship, and structured project incubation may further support translation of ideas into tangible outcomes [60]. Creating peer and interdisciplinary networks that connect students, faculty, and clinical professionals may also facilitate knowledge exchange and collaboration [61]. Finally, clearer progression routes—such as pathways into nursing informatics, data-driven nursing, or interdisciplinary innovation projects—may support students’ transition from learners to emerging innovators.

Based on these findings, a structured and stage-based sequence of AI-related learning opportunities may support more coherent and developmentally aligned learning trajectories within undergraduate nursing curricula. Early learning can focus on foundational concepts, ethical awareness, and relevance to nursing practice, while intermediate learning may emphasize scaffolded hands-on technical training. For senior students, advanced learning opportunities—such as AI-supported clinical decision-making, data literacy, and interdisciplinary innovation projects—may help support the transition from knowledge acquisition to professional identity formation. Incorporating simulation, case-based learning, and collaborative projects with clinical partners may also enhance authenticity and applicability, creating a more coherent pathway aligned with students’ evolving needs and the demands of intelligent healthcare systems.

Although participants were recruited from four universities to enrich experiential variation, this study did not aim to compare differences across institutions, academic years, or levels of prior AI exposure. Rather, the purpose was to explore the overall learning journey of undergraduate nursing students and identify common stage-based patterns across their AI learning experiences. Given the exploratory qualitative design and relatively small sample, in-depth subgroup analysis was beyond the scope of the present study and might have risked overinterpretation. Future research with larger and more contextually diverse samples may further examine how institutional, curricular, and year-level factors shape undergraduate nursing students’ AI learning experiences.

## Conclusion

This study applied a journey mapping approach to examine undergraduate nursing students’ learning experiences with artificial intelligence across four distinct stages, highlighting key barriers, emotional transitions, and evolving educational needs. By adopting a student-centered and process-oriented perspective, this exploratory study provides a more nuanced understanding of how nursing students experience, interpret, and adapt to AI learning over time, and enriches current discussions on AI education in nursing.

## Supplementary Information

Supplementary Material 1.

Supplementary Material 2.

## Data Availability

The qualitative data generated and analyzed during the current study are not publicly available due to confidentiality and privacy considerations but are available from the corresponding authors on reasonable request.
