# Supplementary material for: Understanding undergraduate nursing students’ learning journeys with artificial intelligence: a journey mapping study
Source: BMC Med Educ. 2026 Apr 23;26:930. doi: 10.1186/s12909-026-09271-y (PMC13238069; doi:10.1186/s12909-026-09271-y)
Supplement: Supplementary file 1 — Supplementary Material 1. [file 12909_2026_9271_MOESM1_ESM.docx]

**Supplementary File 1. Semi-Structured Interview Guide**

**Title**

**A Journey Mapping Study of Undergraduate Nursing Students’ Learning Experiences with Artificial Intelligence**

**Description**

This supplementary file provides the complete semi-structured interview guide used in the study “Mapping the Learning Journey of Undergraduate Nursing Students in Artificial Intelligence: Barriers and Pathways for Educational Optimization”. The guide was developed based on the study aims, relevant literature, and preliminary observations of learning contexts. It was reviewed by two experts in nursing education and pilot-tested with two non-participant students to ensure clarity and relevance.

This guide corresponds to the methodology described in Section 2.4: Development of the Interview Guide.

**Instructions for Use**

Interviewers used the guide flexibly during data collection. Probing, clarifying, and follow-up questions were added where necessary to encourage elaboration and to explore emerging issues in depth. Questions 6 and 7 were asked only to senior-year students who had entered clinical practice.

**Semi-Structured Interview Questions**

**1. Initial Perceptions and Prior Understanding**

1.1 Before formally studying AI-related courses, how did you perceive or imagine AI in the context of nursing practice?
1.2 What sources or experiences influenced your early impressions of AI?

**2. Learning Activities and Engagement**

2.1 From your first exposure to AI concepts until now, what kinds of learning activities or explorations have you engaged in?
2.2 What motivated or discouraged you during these activities?

**3. Cognitive and Practical Challenges**

3.1 What were the main challenges or obstacles you encountered when learning AI concepts, theories, or tools?
3.2 Could you share a specific example that made you feel confused, frustrated, or challenged?

**4. Connecting AI with Nursing Practice**

4.1 Have you attempted to connect AI knowledge with nursing or clinical scenarios?
4.2 What difficulties did you encounter when trying to make these connections?

**5. Emotional Experiences During Learning**

5.1 During your AI learning journey, at which moments did you feel the strongest sense of achievement, confusion, or frustration?
5.2 What contributed to these feelings?

**6. Clinical Exposure to AI (For students with clinical experience only)**

6.1 Have you observed or used AI technologies during clinical observation or internship?
6.2 If so, how did these experiences shape your understanding of AI in nursing practice?

**7. Interpretation of AI–Clinical Discrepancies (For students with clinical experience only)**

7.1 When AI-generated outputs or alerts differed from actual clinical observations or patient conditions, how did you interpret and respond to these discrepancies?
7.2 What did these moments make you think about the role of AI in clinical decision-making?

**8. Influence on Future Career Development**

8.1 How do you think AI may influence your future career development in nursing?
8.2 Does this bring motivation, pressure, or uncertainty for you?

**9. Engagement in AI-Related Innovation or Projects**

9.1 Have you participated in any AI-related projects, innovations, or explorations beyond course requirements?
9.2 What prompted or supported your participation?

**10. Learning Support and Resource Needs**

10.1 When encountering difficulties during AI learning, what types of support did you seek?
10.2 How could teachers, courses, or institutions better support your learning needs?

**11. Reflection on the Learning Journey**

11.1 Looking back on your overall AI learning experience, what do you consider the key turning points or influential factors in your journey?
11.2 What advice would you give to future nursing students beginning to learn AI?
